# A dead giveaway: Foraging vultures and other avian scavengers respond to auditory cues

**DOI:** 10.1002/ece3.6366

**Published:** 2020-05-19

**Authors:** Craig R. Jackson, Thomas Maddox, Franco P. Mbise, Bård G. Stokke, Jerrold L. Belant, Kjetil Bevanger, Sarah M. Durant, Robert Fyumagwa, Peter S. Ranke, Eivin Røskaft, Roel May, Frode Fossøy

**Affiliations:** ^1^ Norwegian Institute for Nature Research Trondheim Norway; ^2^ Fauna and Flora International Cambridge UK; ^3^ University of Dodoma Dodoma Tanzania; ^4^ College of Environmental Science and Forestry State University of New York Syracuse NY USA; ^5^ Institute of Zoology Zoological Society of London London UK; ^6^ Tanzanian Wildlife Research Institute Arusha Tanzania; ^7^ Norwegian University of Science and Technology Trondheim Norway

**Keywords:** birds of prey, carrion, optimal foraging, raptors, scavengers, sensory cues

## Abstract

Carrion represents an unpredictable and widely distributed primary food source for vultures and other avian scavengers. Avian scavengers in African savanna ecosystems are reported to rely exclusively on visual stimuli to locate carcasses. However, carnivores’ predation of large mammalian herbivores and subsequent competition for access to the carcass can result in considerable noise, often audible over long distances and for prolonged periods. Vultures and other avian scavengers may therefore detect and respond to these auditory cues, as do the mammalian carnivores alongside which vultures have coevolved, but this has not been investigated to date. Working in the Serengeti ecosystem, Tanzania, we used diurnal auditory broadcasts to simulate predation and competitive carnivore feeding interactions. Based on the current understanding of avian scavenger ecology, we hypothesized that avian responses to call‐in stations would be evoked exclusively by visual, rather than auditory, cues. We therefore predicted that (a) the arrival of avian scavengers at call‐in stations should be preceded and facilitated by mammalian carnivores and that (b) the arrival of avian scavengers would be positively correlated with the number of mammalian scavengers present, which would increase detectability. We recorded 482 birds during 122 separate playback events. In 22% of these instances, avian scavengers arrived first, ruling out responses based exclusively on visual observations of mammalian carnivores, thereby contradicting our first prediction. Furthermore, the first avian arrivals at survey sessions were inversely related to the number of hyenas and jackals present, contradicting our second prediction. Since no bait or carcasses were used during the experiments, these responses are indicative of the birds’ ability to detect and respond to audio stimuli. Our findings challenge the current consensus of sensory perception and foraging in these species and provide evidence that avian scavengers have the ability to use sound to locate food resources.

## Graphical abstract

Avian scavengers in African savanna ecosystems are reported to rely exclusively on visual stimuli to locate carcasses. Here, we report on the response of vultures and other avian scavengers to auditory cues indicative of foraging opportunities. Based on our findings, we hypothesize that these birds are able to utilize auditory cues while foraging.

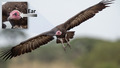

## 1 INTRODUCTION

Foraging strategies form a central part of animal ecology, influencing organisms’ movement, behavior, and ultimately their fitness (Spiegel, Getz, & Nathan, 2013). Although cues used by animals to indicate potential foraging opportunities vary considerably among taxa and environments, successful detection thereof is vital to maximize foraging success. As a result, natural selection has shaped foraging strategies and animals’ physical, physiological, or neurological adaptations to increase search efficiency and food acquisition rates (Preston, Pitchford, & Wood, 2010). In birds, many species rely on visual and/or olfactory cues while foraging (Goldsmith, 1990; Martin, 2017; Nevitt & Bonadonna, 2005; Potier, Duriez, Célérier, Liegeois, & Bonadonna, 2019). Although sound is important in the behavioral ecology of many birds (e.g., mate finding, territorial displays), few species reportedly use auditory cues during foraging (e.g., Wagner, Kettler, Orlowski, & Tellers, 2013; Onrust et al., 2017).

Avian dietary specialization differs considerably, however, as can be illustrated by the vultures inhabiting African savanna ecosystems. Unlike species of New World vultures (Cathartidae) (Potier et al., 2019), Africa's vultures (Accipitridae) reportedly have no refined olfactory senses (Houston, 1985). However, closely related species are able to use olfaction during foraging (Gilbert & Chansocheat, 2006; Nelson Slater & Hauber, 2017; Potier, 2019), indicating that it might be possible for the African species too. Current consensus is that they locate food exclusively by sight (Martin, Portugal, & Murn, 2012; Mundy, Bunchart, Ledger, & Piper, 1992; Spiegel et al., 2013). This may be achieved, for example, through direct observation of carcasses or by observing the behavior of conspecifics, other avian scavengers, or mammalian carnivores that may indicate carrion availability (Jackson, Ruxton, & Houston, 2008; Kane, Jackson, Ogada, Monadjem, & McNally, 2014). Other avian scavengers include Bateleur (*Terathopius ecaudatus*), Tawny (*Aquila rapax*), and Steppe (*A. nipalensis*) eagles which may play an important facilitatory role when followed by vultures to carcasses (Kane et al., 2014; Kane & Kendall, 2017).

Here, we describe the responses of avian scavengers to diurnal audio broadcasts which were conducted to survey mammalian carnivores in the Serengeti ecosystem, Tanzania. We broadcast auditory cues to mimic terrestrial predator–prey interactions indicative of potential foraging opportunities. No carcasses or bait were deployed, so the auditory cues were the only indicator of potential food availability. Based on current understanding of avian scavenger ecology, we hypothesized that avian scavengers would not respond based on auditory cues alone. We predicted that (a) the arrival of avian scavengers at call‐in stations should be preceded and facilitated by mammalian carnivores. Furthermore, we predicted that (b) the arrival of avian scavengers would be positively correlated with the number of mammalian scavengers, since the more mammalian scavengers present, the easier it should be to detect the foraging opportunity. Finally, potential facilitation among the avian scavenger species was quantified.

## 2 METHODS

Call‐in stations sessions were conducted in the Serengeti Ecosystem (Serengeti National Park, Ngorongoro Conservation Area, and Loliondo Game Controlled Area) to survey large carnivore populations between July 1999 and April 2001 (Maddox, 2003) and, replicating the same survey, during November 2016 to July 2017. Individual call‐in station sites were usually located at least 10 km distant from each other and resurveyed ≥3 months apart. Call‐in station playbacks were initiated between 06:00 and 09:00 hr using a 15‐min audio track that consisted of 3 min of a wildebeest (*Connochaetes taurinus*) calf in distress and 12 min of spotted hyenas (*Crocuta crocuta*) and lions (*Panthera leo*) competing over a kill. This was broadcast four times, for a total of 60 min (*n* = 318 sessions), using powerful loudspeakers. A sound meter was used to ensure that the sound was consistently broadcast at a peak sound pressure of 114 db. For detailed methodology, see Maddox (2003).

Avian responses to playbacks were defined by birds’ targeted approach toward the sound source, which frequently included approaches to <100 m of the speakers, as well as landing or perching in close proximity. We recorded the number and species of major avian scavengers that were recorded at >5 independent call‐in stations. Species recorded included Ruppell's griffon vulture (*Gyps rueppelli*), white‐headed vulture (*Trigonoceps occipitalis*), lappet‐faced vulture (*Torgos tracheliotos*), hooded vulture (*Necrosyrtes monachus*), white‐backed vulture (*Gyps africanus*), bateleur eagle (*Terathopius ecaudatus*), black crow (*Corvus capensis*), black kite (*Milvus migrans*), marabou stork (*Leptoptilos crumenifer*), tawny eagle, and steppe eagle. Given their morphological, ecological, and behavioral similarities, data for tawny and steppe eagles were pooled.

**FIGURE 1:**
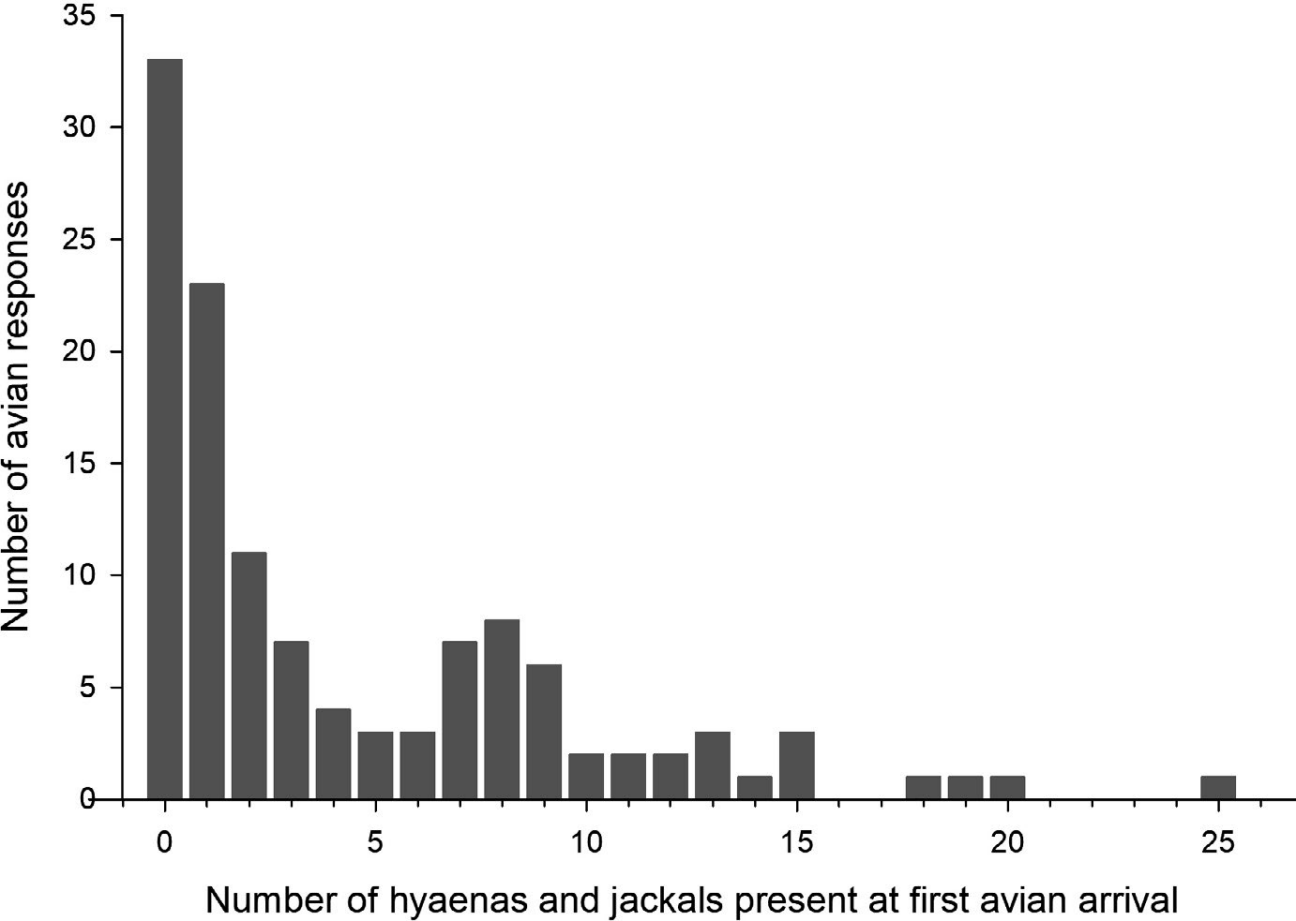
The total number of spotted hyenas and black‐backed and golden jackals at individual call‐in stations upon the arrival of the first avian scavenger. The number of avian responses (sessions) are shown in bars (*n* = 122 avian scavenger arrivals at independent call‐in station sessions)

To assess interactions and potential variability in species’ facilitatory roles within the avian scavenger guild, we made use of a widely used measure of dominance, David's score (David, 1987). This approach tracks individuals dominating in intraspecific interactions between multiple individuals (Poisbleau, Jenouvrier, & Fritz, 2006). In the present application of the David's score methodology, both the variety and the arrival order of the different avian species were incorporated into the calculations, such that the results provide a “facilitation score.” Since facilitation by carnivores did not appear important (see Figure 1), as has been reported elsewhere (Kane & Kendall, 2017), avian arrival order was assessed without considering whether mammalian carnivores were present or not.

David's Score was calculated using the formula:

$$\text{DS}=w+w_{2}-l-l_{2}$$

where *w* represents the sum of *i*'s *P_ij_* values, *w*_2_ represents the summed *w* values (weighted by the appropriate *P_ij_* values) of those individuals with which *i* interacted, *i* represents the sum of i's *P_ji_* values and *l*_2_ represents the summed *i* values (weighted by the appropriate *P_ji_* values) of those individuals with which *i* interacted. The proportion of wins by individual *i* in his interactions with another individual *j* (*P_ij_*) is the number of times that *i* defeats *j* (*α_ij_*) divided by the total number of interactions between *i* and *j* (*n_ij_*), that is, *P_ij_* = *α_ij_*/*n_ij_* (Gammell, de Vries, Jennings, Carlin, & Hayden, 2003).

## 3 RESULTS AND DISCUSSION

We detected a total of 320 vultures, 109 eagles, 21 storks, 16 kites, and 16 crows at 38.4% (122 of 318) of call‐in station sessions (Table 1). In 87.6% of these sessions, birds landed or perched at the survey site. Five of the six vulture species known to occur in the study area appeared at call‐in stations; only the Egyptian vulture (*Neophron percnopterus*) went undetected.

**TABLE 1:** The response of avian scavenger species to 318 call‐in station sessions in the Serengeti ecosystem

	Number of individuals	Number of separate sessions	Arrived first (number of sessions)
Ruppell's griffon vulture	14	8	0
White‐headed vulture	18	10	1
Lappet‐faced vulture	78	33	6
Hooded vulture	82	30	4
White‐backed vulture	128	30	3
Tawny/Steppe eagle	101	69	10
Bateleur	8	8	0
Marabou	21	7	1
Black kite	16	10	1
Black crow	16	8	1
**Total**	**482**	**213**	**27**

Note

For each species, information on the number of individuals (total number detected at all sessions), number of separate sessions (number of sessions at which ≥1 individual was detected) and the number of sessions at which a species arrived first (before any other mammalian or avian scavengers), is presented.

Our first prediction, that the response and arrival of avian scavengers to call‐in stations should be facilitated and preceded by the presence of mammalian carnivores, was not supported. Despite the typically rapid arrival of hyenas and/or jackals (black‐backed jackals (*Canis mesomelas*) and golden jackals (*C. aureus*/*anthus*)) to 90.1% (289 of 318) of all call‐in station sessions, all avian scavengers except for Ruppell's griffon vulture, were recorded arriving first, before any other mammal or other bird species (Table 1). Avian scavengers were the first animals to arrive at 22.1% (27 of 122 sessions) of call‐in station sessions that attracted birds. In the absence of any visual and olfactory cues (no carcass or mammalian scavengers), sound was the only potential cue that might have elicited this behavioral response.

Our second prediction of a positive numerical response at call stations between avian scavengers and mammalian carnivores was also not supported. Since spotted hyenas and jackals arrived at most call‐in stations, we assessed the relationship between avian scavenger arrival and the total number of hyenas and jackals already present when the first bird(s) arrived. We found that the arrival of the first avian scavenger was most likely to occur in the absence of these carnivores and, overall, the avian response rate was inversely correlated with the number of hyenas and jackals already present (Spearman rank order correlation, *r* = −0.895, *p* < .001; Figure 1). Similarly, Kane and Kendall (2017) found that in the Maasai Mara National Reserve, Kenya, scavenging birds arrived first and facilitated mammalian scavengers, rather than vice versa. Facilitation of avian scavengers by mammalian carnivores did therefore not appear important.

**FIGURE 2:**
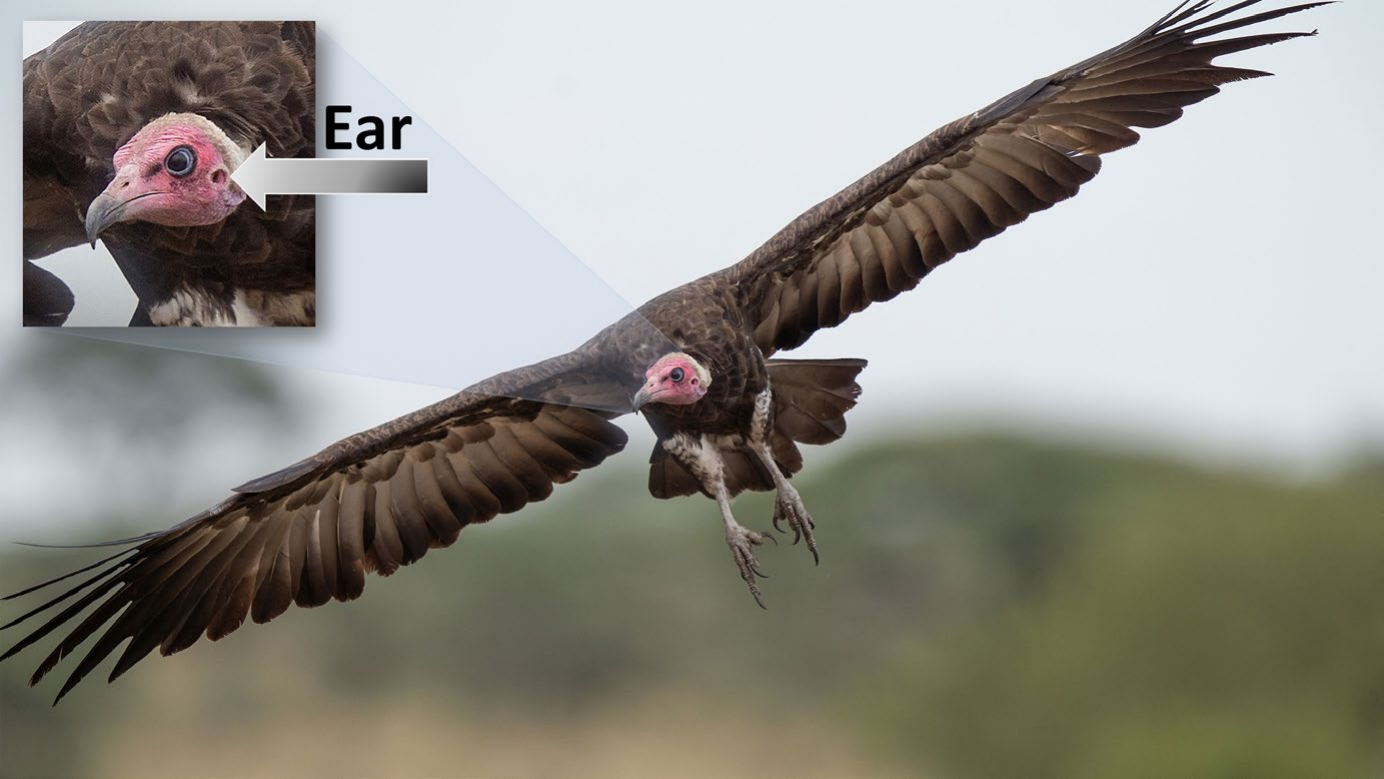
More to it than meets the eye: the ear. A hooded vulture coming in to land, its large eye and ear clearly visible. Although the role of auditory cues in the foraging ecology of avian scavengers has been disregarded to date, we recorded numerous responses to our playback surveys where sound was the only possible cue upon which these birds could act. Photo: Per Harald Olsen

Vultures are near obligate scavengers and have evolved alongside the large carnivores whose predatory behaviors can contribute to their foraging success. The capture and killing of large mammalian prey species is frequently noisy, can be heard over long distances, and can take considerable time, during which the dying animal may emit distress calls. Following the active predation phase, intra‐ and interspecific competition among carnivores can result in prolonged periods of foraging‐related noise, particularly by hyenas (which may be facilitatory, attracting other clan members to a kill). Mammalian carnivores readily respond to such auditory cues and audio playbacks consequently serve as a recognized carnivore survey technique (Cozzi, Broekhuis, McNutt, & Schmid, 2013). Birds have the most evolved hearing ability among nonmammalian vertebrates (Necker, 2000), and their auditory perception is similar to that of mammals (Dooling, Leek, Gleich, & Dent, 2002). Since vultures have evolved alongside the evolutionary adaptations of mammalian predators and scavengers, it is not surprising that they have developed the ability to detect and respond to these reliable auditory cues (Figure 2).

In 36.1% of sessions (44 of 122), two or more avian species arrived at the same session, providing support for avian social facilitation (Kane et al., 2014; Kane & Kendall, 2017; Kendall, 2013). Selection shapes mechanisms increasing scavengers’ foraging efficiency, particularly the detection of spatially dispersed and temporally ephemeral food items (Jackson et al., 2008). Strategies extend beyond physical and physiological specializations, with sociality and the associated benefits derived from social foraging behavior hypothesized as a mechanism permitting vultures to meet energy requirements (Jackson et al., 2008). “Public information” obtained through the observation of other avian scavengers, including conspecifics, is likely a key foraging cue for vultures (Dermody, Tanner, & Jackson, 2011; Jackson et al., 2008; Kane et al., 2014). While the visibility of a carcass or feeding carnivores can be restricted by vegetation and topography, observations of airborne birds are not. With their large wingspans (up to 2.5 m) and no visual obstructions, vultures and eagles in flight are detectable at considerably longer distances than are carcasses, and collective search efforts increase the total area surveyed for food.

Assessing variability in species’ potential facilitatory roles using the David's Score metric (Table 2) revealed that although the tawny/steppe eagles were the most numerous first responders (51 sessions), no subsequent avian arrivals were recorded in 72.5% (*n* = 37) of these sessions. Consequently, the eagles only received a moderate facilitation score given the comparatively low number of competitive interactions that could be scored (i.e., a lack of facilitation). Hooded and lappet‐faced vultures received the highest scores; during 21 sessions each of these species arrived before any other birds (i.e., 42 sessions in total) and in more than half of these occasions were followed by other avian scavengers (25 of 42 sessions).

**TABLE 2:** Avian scavenger facilitation hierarchies, calculated using David's score (DS) and based on species‐specific arrival information (number of individuals, number of sessions, arrival order), irrespective of the presence or absence of mammalian scavengers

	Number of sessions			Dominance rankings	
	First avian responder	Arrived after another avian species	Alone	DS rank (DS value)	Dominance as facilitator
Ruppell's griffon vulture	1	7	1	10 (−11.365)	Low
White‐headed vulture	4	6	2	9 (−10.38)	Low
Lappet‐faced vulture	21	12	6	2 (10.22)	High
Hooded vulture	21	9	11	1 (14.12)	High
White‐backed vulture	8	22	3	6 (−3.78)	Medium
Tawny/Steppe eagle	51	16	37	5 (3.19)	Medium
Bateleur	6	2	3	3 (7.935)	High
Marabou	2	5	1	7 (−6.54)	Low
Black kite	5	5	2	4 (4.22)	Medium
Black crow	3	5	2	8 (−7.62)	Low

By comparison, the other three vulture species only arrived first in a total of 13 sessions. While variability in species density is likely to influence this, temporal segregation occurs among avian scavengers and time of day and may too be a particularly important factor (Kendall, 2014). Our call‐in stations were conducted soon after sunrise, when ambient temperatures were still relatively cool. The hotter periods of the day are associated with greater thermal updrafts, and optimal soaring conditions typically occur between *ca*. 11:00 and 16:00 (Pennycuick, 1973). Avian scavenger species with heavier wing loads, that is, body mass to wing surface area ratio, are unable to soar during the cooler morning hours (Kendall, 2014; Pennycuick, 1973). Pennycuick (1973) assessed wing loading values in ten species of soaring birds, all of which occur in the Serengeti ecosystem and found that two groups emerged: a light wing loading group and heavier wing loading group. The four species included in the light wing loading group were the tawny eagle, hooded vulture, white‐headed vulture, and the lappet‐faced vulture. Although we included data on 10 different bird species, these four light wing loading species were the first avian arrivals at 79.5% (97 of 122) of the sites. Although species‐specific variability in density, distribution, and habitat preferences are likely to influence avian scavenger arrival characteristics, the early morning timing of audio playbacks were possibly better suited to attract birds with lower wing loads, as these species are able to remain airborne on the weakest of early morning thermals (Pennycuick, 1973).

Since both our predictions were not supported, we conclude that foraging avian scavengers are able to detect and respond to auditory cues. Our call‐in station surveys aimed to quantify large carnivore population densities, but the responses of avian scavengers have provided additional insights into avian behavioral ecology. Although playback surveys represent a standard carnivore survey technique, they are typically conducted at night when lions and hyenas (focal species) are most active (Cozzi et al., 2013). Our surveys in the Serengeti, where daytime predation by usually nocturnal predators such as lions and hyenas is not uncommon, were initiated between 06:00 and 09:00 hr which made it possible to attract and record the regular response of vultures and eagles which would not have occurred at night. Despite several decades of research, this elementary component of their natural history has not yet been recognized, and based on our findings, we hypothesize a potential role for auditory sensory perception in foraging techniques. This could be further tested using a more rigorous experimental design that incorporates experimental controls, unlike our protocol which was designed for surveying carnivores. Such an approach could potentially include the use of an alternative audio track not indicative of foraging opportunities, perhaps combined with visual cues (*cf*. Kane & Kendall, 2017), to comprehensively test avian responses and determine the relative roles played by auditory and faciliatory cues. Such research would contribute to a better understanding of the multiple approaches that avian scavengers, many of which continue to decline perilously, use to locate carrion.

## CONFLICT OF INTEREST

None declared.

## AUTHOR CONTRIBUTION

**Craig R. Jackson:** Conceptualization (lead); Formal analysis (lead); Funding acquisition (equal); Investigation (equal); Methodology (equal); Project administration (equal); Writing‐original draft (lead). **Thomas Maddox:** Conceptualization (lead); Data curation (lead); Formal analysis (equal); Funding acquisition (equal); Investigation (lead); Methodology (equal); Project administration (equal). **Franco P. Mbise:** Data curation (equal); Investigation (lead); Methodology (equal); Writing‐review & editing (equal). **Bård G. Stokke:** Conceptualization (equal); Funding acquisition (equal); Investigation (equal); Project administration (equal); Supervision (equal); Writing‐review & editing (lead). **Jerrold L. Belant:** Conceptualization (equal); Investigation (equal); Methodology (equal); Project administration (equal); Supervision (lead); Writing‐review & editing (lead). **Kjetil Bevanger:** Funding acquisition (equal); Investigation (equal); Project administration (equal); Supervision (equal); Writing‐review & editing (equal). **Sarah M. Durant:** Conceptualization (equal); Funding acquisition (lead); Investigation (lead); Methodology (equal); Project administration (equal); Supervision (equal); Writing‐review & editing (lead). **Robert Fyumagwa:** Funding acquisition (equal); Investigation (supporting); Project administration (equal); Supervision (equal); Writing‐review & editing (equal). **Peter S. Ranke:** Investigation (supporting); Writing‐review & editing (equal). **Eivin Røskaft:** Funding acquisition (lead); Project administration (equal); Supervision (equal); Writing‐review & editing (equal). **Roel May:** Conceptualization (equal); Formal analysis (lead); Funding acquisition (equal); Investigation (equal); Methodology (equal); Project administration (equal); Supervision (equal); Writing‐review & editing (lead). **Frode Fossøy:** Conceptualization (equal); Formal analysis (equal); Funding acquisition (equal); Investigation (equal); Methodology (equal); Project administration (equal); Writing‐review & editing (lead).

## Supporting information

Appendix S1

## Data Availability

The data used and presented in this paper have been included as Appendix S1.

## References

[ece36366-bib-0001] Cozzi, G. , Broekhuis, F. , McNutt, J. , & Schmid, B. (2013). Density and habitat use of lions and spotted hyenas in northern Botswana and the influence of survey and ecological variables on call‐in survey estimation. Biodiversity and Conservation, 22, 2937–2956. 10.1007/s10531-013-0564-7

[ece36366-bib-0002] David, H. A. (1987). Ranking from unbalanced paired‐comparison data. Biometrika, 74, 432–436. 10.1093/biomet/74.2.432

[ece36366-bib-0003] Dermody, B. J. , Tanner, C. J. , & Jackson, A. L. (2011). The evolutionary pathway to obligate scavenging in Gyps vultures. PLoS ONE, 6, e24635 10.1371/journal.pone.0024635 21931786 PMC3169611

[ece36366-bib-0004] Dooling, R. J. , Leek, M. R. , Gleich, O. , & Dent, M. L. (2002). Auditory temporal resolution in birds: Discrimination of harmonic complexes. The Journal of the Acoustical Society of America, 112, 748–759. 10.1121/1.1494447 12186054

[ece36366-bib-0005] Gammell, M. P. , de Vries, H. , Jennings, D. J. , Carlin, C. M. , & Hayden, T. J. (2003). David's score: A more appropriate dominance ranking method than Clutton‐Brock et al'.s index. Animal Behaviour, 66, 601–605. 10.1006/anbe.2003.2226

[ece36366-bib-0006] Gilbert, M. , & Chansocheat, S. (2006). Olfaction in accipitrid vultures. Vulture News, 55, 6–7.

[ece36366-bib-0007] Goldsmith, T. H. (1990). Optimization, constraint, and history in the evolution of eyes. The Quarterly Review of Biology, 65, 281–322. 10.1086/416840 2146698

[ece36366-bib-0008] Houston, D. C. (1985). evolutionary ecology of afrotropical and neotropical vultures in forests. Ornithological Monographs, 36, 856–864. 10.2307/40168321

[ece36366-bib-0009] Jackson, A. L. , Ruxton, G. D. , & Houston, D. C. (2008). The effect of social facilitation on foraging success in vultures: A modelling study. Biology Letters, 4, 311 10.1098/rsbl.2008.0038 18364309 PMC2610049

[ece36366-bib-0010] Kane, A. , Jackson, A. L. , Ogada, D. L. , Monadjem, A. , & McNally, L. (2014). Vultures acquire information on carcass location from scavenging eagles. Proceedings of the Royal Society B: Biological Sciences, 281, 20141072 10.1098/rspb.2014.1072 PMC4173674 25209935

[ece36366-bib-0011] Kane, A. , & Kendall, C. J. (2017). Understanding how mammalian scavengers use information from avian scavengers: Cue from above. Journal of Animal Ecology, 86, 837–846. 10.1111/1365-2656.12663 28295318

[ece36366-bib-0012] Kendall, C. J. (2013). Alternative strategies in avian scavengers: How subordinate species foil the despotic distribution. Behavioral Ecology and Sociobiology, 67, 383–393. 10.1007/s00265-012-1458-5

[ece36366-bib-0013] Kendall, C. J. (2014). The early bird gets the carcass: Temporal segregation and its effects on foraging success in avian scavengers. The Auk, 131, 12–19. 10.1642/AUK-13-201.1

[ece36366-bib-0014] Maddox, T. M. (2003). The ecology of cheetahs and other large carnivores in a pastoralist‐dominated buffer zone. Ph.D. Thesis. University College, London.

[ece36366-bib-0015] Martin, G. R. (2017). The sensory ecology of birds. Oxford, UK: Oxford University Press.

[ece36366-bib-0016] Martin, G. R. , Portugal, S. J. , & Murn, C. P. (2012). Visual fields, foraging and collision vulnerability in Gyps vultures. Ibis, 154, 626–631. 10.1111/j.1474-919X.2012.01227.x

[ece36366-bib-0017] Mundy, P. , Bunchart, D. , Ledger, J. , & Piper, S. (1992). The vultures of Africa. London, UK: Academic Press.

[ece36366-bib-0018] Necker, R. (2000). The avian ear and hearing In Whinow G. C. (Ed.) Sturkie's avian physiology (5th ed., pp. 21–28). Amsterdam, The Netherlands: Elsevier.

[ece36366-bib-0019] Nelson Slater, M. , & Hauber, M. E. (2017). Olfactory enrichment and scent cue associative learning in captive birds of prey. Zoo Biology, 36, 120–126. 10.1002/zoo.21353 28198048

[ece36366-bib-0020] Nevitt, G. A. , & Bonadonna, F. (2005). Sensitivity to dimethyl sulphide suggests a mechanism for olfactory navigation by seabirds. Biology Letters, 1, 303–305. 10.1098/rsbl.2005.0350 17148193 PMC1617144

[ece36366-bib-0021] Onrust, J. , Loonstra, A. J. , Schmaltz, L. E. , Verkuil, Y. I. , Hooijmeijer, J. C. , & Piersma, T. J. I. (2017). Detection of earthworm prey by Ruff Philomachus pugnax. Ibis, 159, 647–656.

[ece36366-bib-0022] Pennycuick, C. J. (1973). The soaring flight of vultures. Scientific American, 229, 102–109. 10.1038/scientificamerican1273-102 4731796

[ece36366-bib-0023] Poisbleau, M. , Jenouvrier, S. , & Fritz, H. (2006). Assessing the reliability of dominance scores for assigning individual ranks in a hierarchy. Animal Behaviour, 72, 835–842. 10.1016/j.anbehav.2006.01.024

[ece36366-bib-0024] Potier, S. (2019). Olfaction in raptors. Zoological Journal of the Linnean Society. 10.1093/zoolinnean/zlz121

[ece36366-bib-0025] Potier, S. , Duriez, O. , Célérier, A. , Liegeois, J.‐L. , & Bonadonna, F. (2019). Sight or smell: Which senses do scavenging raptors use to find food? Animal Cognition, 22, 49–59. 10.1007/s10071-018-1220-0 30367315 PMC6326982

[ece36366-bib-0026] Preston, M. D. , Pitchford, J. W. , & Wood, A. J. (2010). Evolutionary optimality in stochastic search problems. Journal of the Royal Society Interface, 7, 1301–1310. 10.1098/rsif.2010.0090 PMC2894891 20335195

[ece36366-bib-0027] Spiegel, O. , Getz, W. M. , & Nathan, R. (2013). Factors influencing foraging search efficiency: Why do scarce lappet‐faced vultures outperform ubiquitous white‐backed vultures? The American Naturalist, 181, E102–E115. 10.1086/670009 23594555

[ece36366-bib-0028] Wagner, H. , Kettler, L. , Orlowski, J. , & Tellers, P. (2013). Neuroethology of prey capture in the barn owl (*Tyto alba* L.). Journal of Physiology‐Paris, 107, 51–61. 10.1016/j.jphysparis.2012.03.004 22510644

