# Supplementary material for: A dead giveaway: Foraging vultures and other avian scavengers respond to auditory cues
Source: Ecol Evol. 2020 May 19;10(13):6769–74. doi: 10.1002/ece3.6366 (PMC7381568; doi:10.1002/ece3.6366)
Supplement: Supplementary file 1 — Appendix S1 [file ECE3-10-6769-s001.pdf]

Supporting information:

Data used for the manuscript entitled "A dead giveaway: foraging vultures and eagles respond to auditory cues"

Explanation of variables:

1. **Species**
    a. HV = Hooded vulture
    b. LFV = Lappet-faced vulture
    c. RGV = Rüppel's (griffon) vulture
    d. TE/SE = Tawny / Steppe eagle
    e. WBV = White-backed vulture
    f. WHV = White-headed vulture
    g. BAT = Bateleur
    h. MS = Marabou stork
    i. BK = Black kite
    j. BC = Black crow
2. **Call-in site ID:**
    a. The site codes at which call-in stations were undertaken and repeated
3. **First:**
    a. This denotes whether this species, at the specified call-in station session, arrived first (Y) or not (N) – before any other birds or mammals
4. **Month:**
    a. Month of the year during which the specified call-in occurred
5. **Year:**
    a. Year during which the specified call-in occurred
6. **Area:**
    a. Call-in station surveys were conducted in either Serengeti (National Park), Loliondo (Game Controlled Area), or Ngorongoro (Conservation Area).
7. **Total inds**:
    a. The total number of individuals of a given species that arrived during the 60-minute call-in station session
8. **Sum Jackals Hyaenas:**
    a. The number of spotted hyaenas and jackals (black-backed and golden) present **upon arrival** of the first avian species. "NA" denotes "not applicable" in instances where birds were not the first avian species to arrive.

Species	Calling_site_ID	First	Month	Year	Region	Total_inds	Sum_Jackals_Hyenas
BAT	LGCA4	N	Nov	2016	Loliondo	1	2
BAT	CI16	N	May	2000	Loliondo	1	9
BAT	CI72	N	Mar	2000	Serengeti	1	1
BAT	CI29	N	Apr	2000	Serengeti	1	6
BAT	CI21	N	Apr	2000	Serengeti	1	NA
BAT	CI02	N	Feb	2000	Loliondo	1	NA
BAT	CI05	N	Jun	2000	Loliondo	1	3
BAT	CI16	N	Oct	2000	Loliondo	1	4
BC	SNP4	N	Nov	2016	Serengeti	2	NA
BC	SNP5	N	Nov	2016	Serengeti	2	1

BC	SNP8	N	Nov	2016	Serengeti	2	NA
BC	SNP9	N	Nov	2016	Serengeti	2	NA
BC	NCA1	N	Nov	2016	Ngorongoro	1	NA
BC	NCA4	N	Nov	2016	Ngorongoro	1	NA
BC	SNP6	Y	Jul	2017	Serengeti	2	0
BC	SNP8	N	Jul	2017	Serengeti	4	9
BK	SNP5	N	Nov	2016	Serengeti	2	NA
BK	LGCA7	Y	Nov	2016	Loliondo	1	0
BK	NCA5	N	Nov	2016	Ngorongoro	2	9
BK	NCA7	N	Nov	2016	Ngorongoro	2	NA
BK	NCA1	N	Feb	2017	Ngorongoro	3	3
BK	SNP8	N	Feb	2017	Serengeti	1	15
BK	SNP10	N	Feb	2017	Serengeti	1	NA
BK	SNP3	N	Feb	2017	Serengeti	2	11
BK	SNP6	N	July	2017	Serengeti	1	NA
BK	SNP7	N	July	2017	Serengeti	1	NA
HV	MA1	N	Nov	1999	Serengeti	6	4
HV	MA2	N	Nov	1999	Serengeti	5	1
HV	CI37	N	Mar	2000	Loliondo	1	2
HV	CI09	N	May	2000	Loliondo	9	NA
HV	CI12	N	May	2000	Loliondo	2	1
HV	CI08A	N	Apr	2001	Loliondo	2	NA
HV	CI24	N	Apr	2001	Serengeti	7	1
HV	CI27	Y	Apr	2001	Serengeti	2	0
HV	CI31	Y	Apr	2001	Ngorongoro	1	0
HV	CI33	N	Apr	2001	Ngorongoro	4	1
HV	CI37	N	Apr	2001	Ngorongoro	1	1
HV	CI41	N	Apr	2001	Ngorongoro	2	19
HV	CI42	N	Apr	2001	Ngorongoro	2	1
HV	CI03	N	Feb	2001	Loliondo	1	3
HV	CI06	N	Feb	2001	Loliondo	2	NA
HV	CI12	N	Feb	2001	Loliondo	2	NA
HV	CI16	N	Feb	2001	Loliondo	2	1
HV	CI33	N	Feb	2001	Ngorongoro	2	6
HV	CI37	N	Feb	2001	Ngorongoro	1	NA
HV	CI38	N	Feb	2001	Ngorongoro	2	7
HV	CI39	N	Feb	2001	Ngorongoro	2	12
HV	CI40	Y	Feb	2001	Ngorongoro	5	0
HV	CI41	Y	Feb	2001	Ngorongoro	5	0
HV	CI24	N	Jan	2001	Serengeti	4	12
HV	CIV1	N	Mar	2001	Loliondo	2	NA
HV	CI16	N	May	2001	Loliondo	1	NA
HV	CIV6	N	May	2001	Loliondo	1	NA
HV	SNP10	N	Feb	2017	Serengeti	3	NA
HV	SNP9	N	Feb	2017	Serengeti	2	9
HV	NCA8	N	Jul	2017	Ngorongoro	1	2

LFV	MK2	Y	Oct	1999	Serengeti	6	0
LFV	CI21	N	Apr	2000	Serengeti	1	3
LFV	CI02	N	Feb	2000	Loliondo	2	7
LFV	CI03	Y	Feb	2000	Loliondo	1	0
LFV	CI17	N	Feb	2000	Loliondo	2	1
LFV	CI35	N	Feb	2000	Loliondo	1	NA
LFV	CI08	N	Mar	2000	Loliondo	2	1
LFV	CI10	N	Mar	2000	Loliondo	1	NA
LFV	CI11	N	Mar	2000	Loliondo	4	7
LFV	CI37	N	Mar	2000	Loliondo	1	NA
LFV	CI50	Y	Mar	2000	Serengeti	3	0
LFV	CI72	N	Mar	2000	Serengeti	5	NA
LFV	CI02	N	May	2000	Loliondo	1	6
LFV	CI09	N	May	2000	Loliondo	4	1
LFV	CI12	N	May	2000	Loliondo	3	NA
LFV	CI19	N	May	2000	Loliondo	2	1
LFV	CI18	N	Oct	2000	Loliondo	1	NA
LFV	CI06	Y	Apr	2001	Loliondo	2	0
LFV	CI08A	N	Apr	2001	Loliondo	2	5
LFV	CI24	N	Apr	2001	Serengeti	1	NA
LFV	CI39	N	Apr	2001	Ngorongoro	1	8
LFV	CI02	N	Feb	2001	Loliondo	2	0
LFV	CI06	Y	Feb	2001	Loliondo	1	0
LFV	CI12	N	Feb	2001	Loliondo	1	2
LFV	CI41	N	Feb	2001	Ngorongoro	2	NA
LFV	NCA5	N	Nov	2016	Ngorongoro	5	NA
LFV	NCA8	N	Nov	2016	Ngorongoro	3	7
LFV	SNP2	N	Nov	2016	Serengeti	3	NA
LFV	SNP3	N	Nov	2016	Serengeti	4	1
LFV	SNP6	N	Nov	2016	Serengeti	2	NA
LFV	NCA3	N	Feb	2017	Ngorongoro	1	NA
LFV	NCA5	Y	Feb	2017	Ngorongoro	3	0
LFV	SNP10	N	Feb	2017	Serengeti	5	1
MS	SNP1	N	Nov	2016	Serengeti	10	14
MS	SNP6	N	Nov	2016	Serengeti	1	NA
MS	CI11	N	Mar	2000	Loliondo	1	NA
MS	CI40	N	Feb	2001	Ngorongoro	3	NA
MS	CI42	Y	Feb	2001	Ngorongoro	4	0
MS	C12	N	Feb	2001	Loliondo	1	NA
MS	CI16	N	Oct	2000	Loliondo	1	NA
RGV	CI11	N	Mar	2000	Loliondo	1	NA
RGV	CI37	N	Mar	2000	Loliondo	1	NA
RGV	CI09	N	May	2000	Loliondo	1	NA
RGV	NCA5	N	Nov	2016	Ngorongoro	4	NA
RGV	SNP3	N	Nov	2016	Serengeti	1	NA
RGV	NCA8	N	Feb	2017	Ngorongoro	2	1

RGV	SNP10	N	Feb	2017	Serengeti	2	NA
RGV	NCA3	N	Jul	2017	Ngorongoro	2	NA
TE/SE	MA1	N	Nov	1999	Serengeti	1	NA
TE/SE	AE1	N	Oct	1999	Loliondo	2	0
TE/SE	AW3	N	Oct	1999	Loliondo	1	25
TE/SE	AW5	N	Oct	1999	Loliondo	2	2
TE/SE	ER1	N	Oct	1999	Loliondo	1	2
TE/SE	NW3	N	Oct	1999	Loliondo	1	7
TE/SE	CI26	N	Apr	2000	Serengeti	1	8
TE/SE	CI37	N	Aug	2000	Ngorongoro	2	1
TE/SE	CI40	N	Aug	2000	Ngorongoro	1	1
TE/SE	CI41	N	Aug	2000	Ngorongoro	2	15
TE/SE	CI42	N	Aug	2000	Ngorongoro	1	8
TE/SE	CIK1	N	Aug	2000	Ngorongoro	2	15
TE/SE	CIK2	N	Aug	2000	Ngorongoro	1	8
TE/SE	CIO1	N	Aug	2000	Ngorongoro	2	1
TE/SE	CI05	Y	Feb	2000	Loliondo	2	0
TE/SE	CI16	N	Feb	2000	Loliondo	1	NA
TE/SE	CI35	N	Feb	2000	Loliondo	2	NA
TE/SE	CI32	N	Jul	2000	Ngorongoro	1	1
TE/SE	CI33	Y	Jul	2000	Ngorongoro	1	0
TE/SE	CI03	N	Jun	2000	Loliondo	1	0
TE/SE	CI06A	N	Jun	2000	Loliondo	1	0
TE/SE	CI12	N	Jun	2000	Loliondo	1	5
TE/SE	CI19	Y	Jun	2000	Loliondo	1	0
TE/SE	CI06	N	Mar	2000	Loliondo	1	1
TE/SE	CI10	N	Mar	2000	Loliondo	2	5
TE/SE	CI37	Y	Mar	2000	Loliondo	1	0
TE/SE	CI48	N	Mar	2000	Serengeti	1	8
TE/SE	CI50	N	Mar	2000	Serengeti	2	NA
TE/SE	CI06	N	May	2000	Loliondo	1	5
TE/SE	CI08	Y	May	2000	Loliondo	1	0
TE/SE	CI09	N	May	2000	Loliondo	1	NA
TE/SE	CI12	N	May	2000	Loliondo	2	NA
TE/SE	CIV3	N	Nov	2000	Loliondo	1	1
TE/SE	CI01A	N	Oct	2000	Loliondo	1	0
TE/SE	CI03	Y	Oct	2000	Loliondo	1	0
TE/SE	CI09	Y	Oct	2000	Loliondo	1	0
TE/SE	CI16	N	Oct	2000	Loliondo	1	NA
TE/SE	CI39	Y	Oct	2000	Ngorongoro	1	0
TE/SE	CI41	N	Oct	2000	Ngorongoro	1	3
TE/SE	CI24	N	Apr	2001	Serengeti	1	NA
TE/SE	CI35	Y	Feb	2001	Ngorongoro	1	0
TE/SE	CI41	N	Feb	2001	Ngorongoro	1	NA
TE/SE	LGCA4	N	Nov	2016	Loliondo	3	4
TE/SE	LGCA8	N	Nov	2016	Loliondo	3	7

TE/SE	NCA1	N	Nov	2016	Ngorongoro	4	20
TE/SE	NCA2	N	Nov	2016	Ngorongoro	3	2
TE/SE	NCA4	N	Nov	2016	Ngorongoro	2	7
TE/SE	NCA6	N	Nov	2016	Ngorongoro	1	NA
TE/SE	NCA7	N	Nov	2016	Ngorongoro	3	9
TE/SE	NCA9	Y	Nov	2016	Ngorongoro	1	0
TE/SE	SNP1	N	Nov	2016	Serengeti	1	9
TE/SE	SNP2	N	Nov	2016	Serengeti	1	8
TE/SE	SNP3	N	Nov	2016	Serengeti	1	NA
TE/SE	SNP6	N	Nov	2016	Serengeti	1	10
TE/SE	SNP8	N	Nov	2016	Serengeti	3	8
TE/SE	SNP9	N	Nov	2016	Serengeti	1	2
TE/SE	LGCA10	N	Feb	2017	Loliondo	1	8
TE/SE	LGCA8	N	Feb	2017	Loliondo	2	3
TE/SE	NCA1	N	Feb	2017	Ngorongoro	1	NA
TE/SE	NCA5	N	Feb	2017	Ngorongoro	1	NA
TE/SE	SNP10	N	Feb	2017	Serengeti	3	NA
TE/SE	SNP2	N	Feb	2017	Serengeti	3	13
TE/SE	SNP3	N	Feb	2017	Serengeti	1	NA
TE/SE	LGCA4	N	Jul	2017	Loliondo	1	13
TE/SE	LGCA7	N	Jul	2017	Loliondo	1	NA
TE/SE	NCA10	N	Jul	2017	Ngorongoro	1	2
TE/SE	NCA3	N	Jul	2017	Ngorongoro	2	4
TE/SE	SNP7	N	Jul	2017	Serengeti	2	6
TE/SE	SNP9	N	Jul	2017	Serengeti	1	10
WBV	MA1	N	Nov	1999	Serengeti	1	NA
WBV	MK2	N	Oct	1999	Serengeti	3	NA
WBV	CI16	N	Feb	2000	Loliondo	2	18
WBV	CI18	N	Feb	2000	Loliondo	1	NA
WBV	CI35	N	Feb	2000	Loliondo	17	2
WBV	CI11	N	Mar	2000	Loliondo	9	NA
WBV	CI37	N	Mar	2000	Loliondo	4	NA
WBV	CI50	N	Mar	2000	Serengeti	6	NA
WBV	CI08	N	May	2000	Loliondo	1	NA
WBV	CI09	N	May	2000	Loliondo	6	NA
WBV	CI12	N	May	2000	Loliondo	25	NA
WBV	CI19	N	May	2000	Loliondo	5	NA
WBV	CI33	N	Apr	2001	Ngorongoro	1	NA
WBV	CI39	N	Apr	2001	Ngorongoro	1	NA
WBV	CI42	N	Apr	2001	Ngorongoro	2	NA
WBV	CI12	N	Feb	2001	Loliondo	6	NA
WBV	CI33	N	Feb	2001	Ngorongoro	2	NA
WBV	CI37	Y	Feb	2001	Ngorongoro	2	0
WBV	CI41	N	Feb	2001	Ngorongoro	2	NA
WBV	CI24	N	Jan	2001	Serengeti	5	NA
WBV	CI05A	Y	May	2001	Loliondo	1	0

WBV	CI16	N	May	2001	Loliondo	1	3
WBV	CIV6	N	May	2001	Loliondo	1	0
WBV	NCA5	N	Nov	2016	Ngorongoro	3	NA
WBV	NCA6	Y	Nov	2016	Ngorongoro	2	0
WBV	NCA8	N	Nov	2016	Ngorongoro	6	NA
WBV	NCA10	N	Feb	2017	Ngorongoro	1	NA
WBV	SNP10	N	Feb	2017	Serengeti	7	NA
WBV	SNP6	N	Feb	2017	Serengeti	3	11
WBV	LGCA7	N	Jul	2017	Loliondo	2	NA
WHV	CI39	N	Aug	2000	Ngorongoro	1	13
WHV	CI16	N	Feb	2000	Loliondo	2	NA
WHV	CI48	N	Mar	2000	Serengeti	1	NA
WHV	CI18	N	Oct	2000	Loliondo	1	2
WHV	CI34	Y	Oct	2000	Ngorongoro	1	0
WHV	CIV1	N	Mar	2001	Loliondo	1	NA
WHV	NCA5	N	Nov	2016	Ngorongoro	2	NA
WHV	NCA8	N	Nov	2016	Ngorongoro	4	NA
WHV	NCA3	N	Feb	2017	Ngorongoro	3	1
WHV	SNP3	N	Feb	2017	Serengeti	2	NA
